# By targeting apoptosis facilitator BCL2L13, microRNA miR-484 alleviates cerebral ischemia/reperfusion injury-induced neuronal apoptosis in mice

**DOI:** 10.1080/21655979.2021.1898134

**Published:** 2021-03-16

**Authors:** Xindong Liu, Xin Wang, Lijuan Zhang, Yi Zhou, Le Yang, Minghao Yang

**Affiliations:** a Department of Neurology, The Second Affiliated Hospital of Chengdu Medical College, China National Nuclear Corporation 416 Hospital, Chengdu City, Sichuan Province, China; b Department of Cerebrovascular Disease, The Second Affiliated Hospital of Guilin Medical University, Guilin City, Guangxi Zhuang Autonomous Region, China

**Keywords:** MiR-484, bcl2l13, cerebral ischemia/reperfusion injury, neuronal apoptosis

## Abstract

Neuronal apoptosis was considered as one of the main factors of cerebral ischemia/reperfusion injury. Understanding the molecular regulatory mechanism of neuronal apoptosis under the cerebral ischemia/reperfusion injury may provide the novel therapeutic targets for cerebral ischemia/reperfusion injury. However, the molecular regulatory mechanism of neurons fate determination under the cerebral ischemia/reperfusion injury remains poorly understood. This study was aimed to delve into the related molecular mechanism of miR-484 on the regulation of cerebral ischemia/reperfusion injury-induced neuronal apoptosis in mice. In this study, quantitative real-time polymerase chain reaction assays revealed that the expression level of miR-484 was down-regulated in neurons following OGD. Then, CCK8 assay western blot assay, and flow cytometry assay verified that upregulation of miR-484 increased viability and inhibited apoptosis of neurons following OGD. Further bioinformatics methods and dual-luciferase reporter assay were applied together to anticipate and certify the interaction between miR-484 and BCL2L13. Finally, cerebral infarct size assessment and TUNEL staining confirmed that overexpression of miR-484 alleviated cerebral ischemia/reperfusion injury in mice, and overexpression of BCL2L13 could abolish the effect of miR-484-suppressed cell apoptosis. All these results suggested that miR-484 alleviates cerebral ischemia/reperfusion injury-induced neuronal apoptosis in mice by targeting apoptosis facilitator BCL2L13.

## Graphical Abstract

**Figure:**
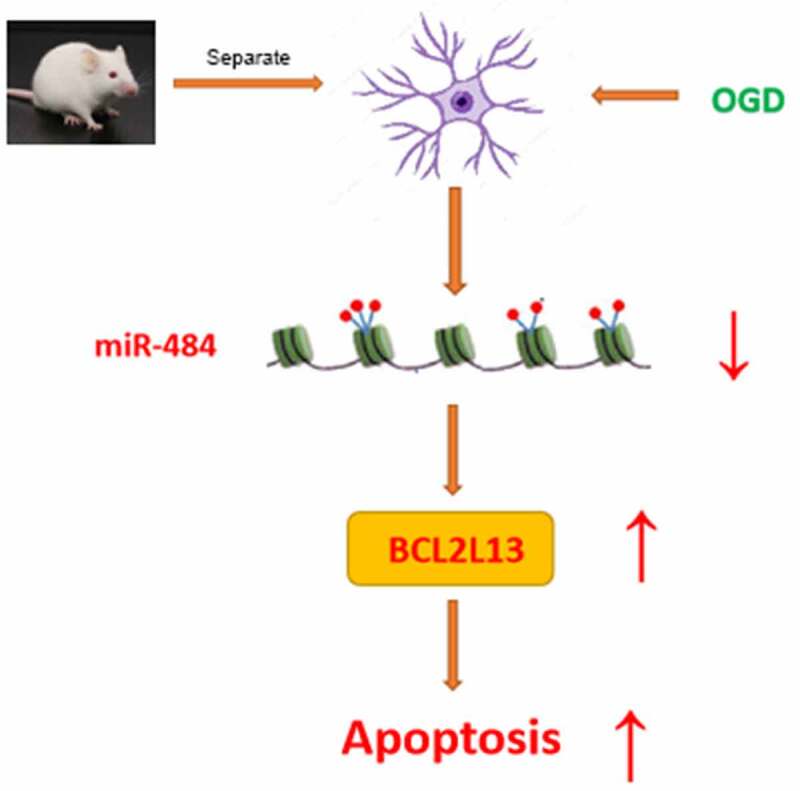

## Introduction

Cerebrovascular disease is one of the diseases with the highest morbidity, mortality, and disability rates in the world. With the improvement of people’s living standards and the change of lifestyle, the incidence of the disease is still on the rise. Cerebrovascular diseases are divided into two categories: hemorrhagic and ischemic cerebrovascular diseases, among which the incidence of ischemic stroke is the highest, accounting for about 75% of all strokes. The most common cause is cerebral atherosclerosis, followed by cerebral artery inflammation. Ischemia penumbral reperfusion around the infarcted tissue is the basis of modern treatment of ischemic stroke, but studies show that reperfusion in the ischemic region aggravates brain tissue damage and leads to more serious brain dysfunction, which is called cerebral ischemia-reperfusion injury (CIR) [1,2]. Cerebral ischemia-reperfusion injury involves a dynamic and complicated pathophysiologic process and is affected by the number of cells inside and outside the physical and chemical factors, which interact with each other between each link and each kind of influence factors. Although in recent years on the pathophysiology of cerebral ischemia and reperfusion injury has made significant progress, but still has not been fully elucidated. With the development of studies, intracellular Ca^2+^ overload, excess production of free radical (FR), cytotoxicity of excitatory amino acid (EAA), inflammatory cascade, acidosis, increased mitochondrial permeability, and apoptosis are considered as the main factors for CIR. Therefore, it is of great theoretical significance and practical value to explore the pathogenesis of cerebral ischemia-reperfusion injury and research and develop drugs to protect against ischemic brain injury.

MicroRNA, as a kind of non-coding RNA, is generally 18–22 bp long. MicroRNA can interact with the 3ʹ untranslated region (UTR) of the gene, thus inhibiting the expression of the gene and playing a variety of important regulatory roles [3]. At present, many studies have revealed the role of microRNAs in CIR and the internal mechanism, providing a direction for the treatment of CIR. Studies have shown that miR-484 can protect myocardial apoptosis during myocardial ischemia-reperfusion injury [4–6]. However, its role and potential mechanism in cerebral ischemia-reperfusion injury are still unknown.

BCL2L13 (Bcl-2-like Protein 13) gene is located in mitochondria and is a kind of mitochondrial outer membrane protein [7]. It was discovered by Kataoka et al. in 2001, and the mRNA expression level in the human heart, pancreas, and placental tissues is relatively high [8]. BCL2L13 has splicing variants, and its gene function changes between promoting apoptosis and inhibiting apoptosis and has positive or negative regulatory effects on apoptosis of different types of cells [9]. As a mitochondrial mitosis receptor [10], BCL2L13 plays a regulatory role in mitochondrial function and also has a significant impact on adipocyte differentiation [11]. In addition, BCL2L13 has also been shown to promote apoptosis in the nervous system [12,13].

Understanding the molecular regulatory mechanism of neuronal apoptosis under the cerebral ischemia/reperfusion injury may provide the novel therapeutic targets for cerebral ischemia/reperfusion injury. This study was aimed to delve into the related molecular mechanism of miR-484 on the regulation of cerebral ischemia/reperfusion injury-induced neuronal apoptosis in mice.

## Materials and methods

### Murine cortical neurons isolation and culture

All animal experiments in this study were in agreement with the Guide for the Care and Use of Laboratory Animals [14] and approved by the Ethics Committee of China National Nuclear Corporation 416 Hospital. Briefly, 12-week-old C57BL/6 mouse embryos were obtained and the whole brains were taken. The cortices were dissected from the brains and mechanically minced. The minced cortical tissues were collected in a tube and dissociated by 0.02% mild trypsinization (Gibco, Carlsbad, CA, USA) at 37°C for 10 min. The murine cortical neurons single-cell suspension was obtained by trituration in 0.004% DNase (Gibco) solution containing 0.05% soybean trypsin inhibitor (Gibco). The cells were resuspended in culture medium (Neurobasal; Gibco) supplemented with% B27 (Gibco), 1% GlutaMAX™ (Gibco), and 1% penicillin/streptomycin (Gibco) and seeded plated on poly-L-lysine-coated 6-well plates at 2.2 × 10^6^ cells. Murine cortical neurons were cultured in a humidified 5% CO_2_ incubator at 37°C [15].

## Oxygen-glucose deprivation (OGD) treatment

To initiate OGD treatment, the primary neurons were washed 3 times and exposed to glucose-free EBSS (Gibco) in a humidified atmosphere with 95% N_2_ and 5% CO_2_ at 37°C. After OGD exposure for 6 hours, EBSS was replaced by a normal neurobasal medium, and murine cortical neurons were kept in the normoxic incubator for 0, 12, 24, and 48 hours [16].

## Quantitative real-time polymerase chain reaction (qPCR)

Total RNA was extracted from cells by using the Trizol reagent (Invitrogen). The quantity and integrity of extracted total RNA were evaluated on a Nano Drop 1000 spectrophotometer (Thermo Fisher Scientific, Inc.). Whole RNA was reversely transcribed into cDNA by using SuperScript III Reverse Transcriptase (Invitrogen) with miRNA RT primer (GenePharma). qRT-PCR was applied by the SYBR Premix EX Taq (Takara, Japan) in the CFX96 Real-Time PCR Detection System (Bio-Rad, Hercules, CA, USA) to detect the expression levels of miR-484. The PCR primers were designed and synthesized by Sangon Biological Engineering Technology (Shanghai, China). U6 small nuclear RNA was used as the endogenous reference genes to normalize miRNA expression levels. The relative expression of miR-484 in each experimental group was analyzed using the 2^−ΔΔCt^ method. All reactions were performed in triplicates. Primer sequences used in this study are shown in Table 1.

**Table 1.** Primers for miR-484 and reference genes

Gene	Primer	Sequence (5ʹ→3ʹ)
miR-484	Forward	CCAGTCGCTTCCTCCAGTAGC
	Reverse	TTCTCTCCTTAGTCCTCGCTC
U6 snRNA	Forward	TCCTCCACGACAACCAAAACC
	Reverse	TCTTTTCCCAAAATCCCAGACTC

## CCK-8 cell viability assay

To assess the extent of cell viability, a CCK-8 assay was used. Briefly, 3.2 × 10^3^ cells were plated into 96-well plates in triplicates and treated in different conditions as indicated in each experiment. Following treatment, 10 μL CCK-8 solution (Beyotime) was applied and incubated for another 4 h. The optical density value of each sample was checked at 450 nm through a microplate reader (BioTek, Winooski, VT, USA).

Briefly, cells were washed in pre-cooling PBS buffer three times, and the total protein was separated by RIPA buffer (Beyotime, Shanghai, China). Protein concentration was detected by using BCA protein assay kits (CoWin Biotechnology). An equal amount of total proteins was electrophoresed to SDS-PAGE. Then, they were transferred to the polyvinylidene difluoride membranes (PVDF; Millipore) with blocked by 5% nonfat milk at room temperature for 0.5 h. The protein was identified by incubated with specific primary antibodies Bax (Rabbit Anti-Bax antibody, ab53154, 1:1500; Abcam, Cambridge, MA, USA), Cleaved Caspase-3 (Rabbit Anti-Cleaved Caspase-3 antibody, ab49822, 1:2000; Abcam), Caspase-3 (Rabbit Anti-Caspase-3 antibody, ab4051, 1:3000; Abcam), BCL2L13 (Rabbit Anti-Bcl2-L-13 antibody, ab25895, 1:3000; Abcam), and β-actin (Rabbit Anti-beta Actin antibody, ab8227, 1:1500; Abcam) overnight at 4°C. Then, the membranes were further incubated with HRP-conjugated goat anti-rabbit immunoglobulin G secondary antibody (ab205718, 1:1500; Abcam) and the bands on the membranes were visualized by the ECL chemiluminescence reagent (Beyotime). The β-actin was used to normalize the amount of the analyzed samples and protein bands were quantified by gray value analysis by ImageJ software (National Institutes of Health).

## Cell Apoptosis

Cell Apoptosis was assessed by Annexin V/FITC and PI apoptosis detection kit (Invitrogen) and flow cytometry (BD Accuri™ C6, USA). Briefly, the cells were digested and resuspended in Annexin V incubation solution. Then, the cells were stained with Annexin V-FITC and PI. The cells were cultured in the dark for 20 min at room temperature and quantified using flow cytometry analysis.

## Cell transfection

The synthetic miR-484 mimics, miR-484 inhibitor, NC mimics (control), and NC inhibitor (control) were acquired from GenePharma (Shanghai, China). After cells were cultured in 6-well plates for 24 h, miR-484 mimics, miR-484 inhibitor, NC mimics (control), and NC inhibitor (control) were transfected into cells by Lipofectamine 2000 (Invitrogen).

## Dual-luciferase reporter assay

The fragments of 3ʹ-UTRs of BCL2L13 mRNA containing the wild-type (WT) and the mutant-type (MUT) binding sites of miR-484 anticipated by using TargetScan were cloned into pmirGLO luciferase report vector (Promega, Madison, WI, USA). The constructed luciferase reporters were called BCL2L13-WT and BCL2L13-MUT, respectively. For luciferase assay, miR-484 mimics, miR-484 inhibitor, NC mimics (control), and NC inhibitor (control) were co-transfected with reporter plasmids into cells by Lipofectamine 2000 (Invitrogen). The luciferase activities were tested by the dual-luciferase kit (Promega) after 2-day transfection.

## Intracerebroventricular injection of lentiviral miR-484

Eighty 12-week-old male C57BL/6 mice (25–30 g) mice were randomly divided into four groups, including sham group, I/R group, I/R + lenti-NC group, and I/R + lenti-miR484 group, with 20 mice in each group. MiR-484 expression was detected in eight mice 7 days after lentivirus overexpression (n = 8), and I/R assay was performed in the remaining mice (n = 12). Lentiviral miR-484 and lentiviral NC (control) vectors were acquired from GenePharma (Shanghai, China). The miR-484 sequence was subcloned into the pLV/H1/GFP vectors. 7 μL lentiviral vector (10^9^ TU·mL^−1^) was incubated at 37°C for 15 min and performed on mice via intracerebroventricular injection for at least 20 min [17].

## Cerebral infarct size assessment

Cerebral Infarct size was measured 24 h after transient middle cerebral artery occlusion (tMCAO), and the mice were sacrificed and perfused with pre-cooling PBS buffer via the ascending aorta. Brains were rapidly removed and sectioned coronally into six sections. The brain slices were stained with 2% 2,3,5-triphenyltetrazolium chloride solution (TTC, Sigma-Aldrich) at 37°C for 15 min followed by fixation with 4% paraformaldehyde for 1 day. The infarct size was quantified with ImageJ software (National Institutes of Health). The infarct size was normalized by the non-ischemic hemisphere and expressed as a percentage of the total brain volume to exclude the effect of cerebral edema [18].

## TUNEL staining

The brain slices were first stained with TUNEL staining (In Situ Apoptosis Detection Kit; Sigma-Aldrich, red) with followed by DAPI staining (Sigma-Aldrich, blue), according to the manufacturer’s protocol.

## Statistical analysis

All data are shown as the mean ± standard error of the mean from three independent experiments. Comparisons between the two groups were performed using Student’s *t*-test. *p* values of <0.01 (two-tailed) were considered to indicate a statistically significant difference. GraphPad Prism 5 (GraphPad Software, Inc.) was used for analysis.

## Results

**The expression level of miR-484 was down-regulated in neurons following oxygen-glucose deprivation (OGD).**

**Figure 1.**
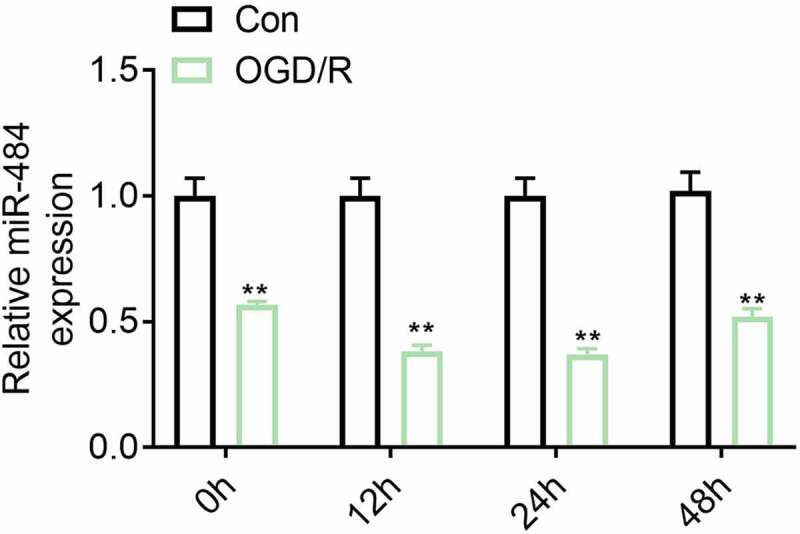
**The expression level of miR-484 was down-regulated in neurons following oxygen-glucose deprivation (OGD)**. The mRNA expression levels of miR-484 were detected in OGD treated neurons. Data were presented as the mean ± SD with three independent experiments. **p < 0.01

Studies have shown that miR-484 can protect myocardial apoptosis during myocardial ischemia-reperfusion injury. However, its role and potential mechanism in cerebral ischemia-reperfusion injury are still unknown. This study was aimed to delve into the related molecular mechanism of miR-484 on the regulation of cerebral ischemia/reperfusion injury-induced neuronal apoptosis in mice. To investigate the correlation between the expression levels of miR-484 and neurons in cerebral ischemia/reperfusion injury, murine cortical neurons were treated by OGD and reoxygenation for varying degrees followed by the detection of miR-484 expression level by qRT-PCR. The result indicated that the expression level of miR-484 was down-regulated in neurons following ODG (Figure 1). This result hinted that miR-484 may be involved in the regulation of the mechanism of neurons in cerebral ischemia/reperfusion injury.

## MiR-484 increased viability and inhibited apoptosis of neurons following OGD

**Figure 2.**
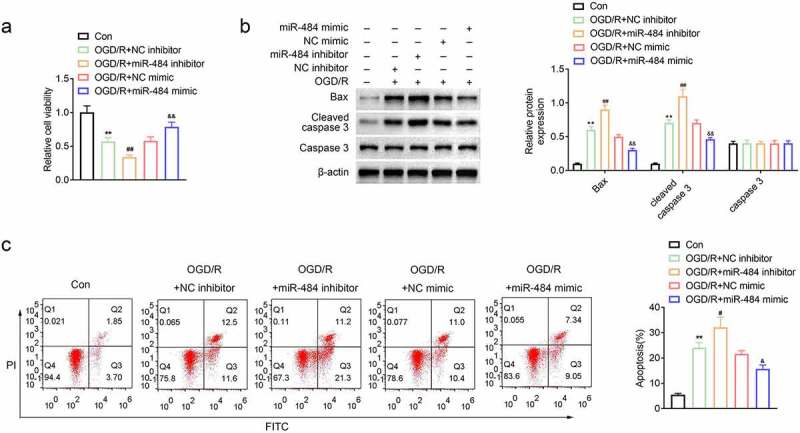
**MiR-484 increased viability and inhibited apoptosis of neurons following oxygen-glucose deprivation (OGD)**. (a) CCK8 assay was applied to determine cell viability for each group, including OGD treated group, OGD treated + miR-484 inhibitor group, OGD treated + NC inhibitor group, OGD treated + miR-484 mimics group, and OGD treated + NC mimics group. (b) Western blot was applied to detect cell apoptosis-related proteins for each group. (c) Flow cytometry analysis was applied to detect apoptosis for each group. Data were presented as the mean ± SD with three independent experiments. **p < 0.01 versus control group, ^#^*p* < 0.05 and ^##^*p* < 0.01 versus OGD treated + NC inhibitor group, and ^&^*p* < 0.01 and ^&&^*p* < 0.01 versus OGD treated + NC mimics group

To explore the role of miR-484 in the survival and apoptosis of OGD-treated neurons, murine cortical neurons were transfected with miR-484 inhibitor, NC inhibitor (control), miR-484 mimics, and NC mimics (control), respectively. Then, cell viability was assessed in each group, and the results revealed that the cell viability of neurons decreased obviously after OGD treatment. Meanwhile, the cell viability of neurons was further reduced compared to the NC inhibitor control group while neurons were transfected with the miR-484 inhibitor. However, the cell viability of neurons was enhanced compared to the NC mimics control group while neurons were transfected with the miR-484 mimics (Figure 2a). In addition, cell apoptosis-related proteins were also examined by western blot in each group, and the results confirmed that OGD treatment and miR-484 inhibitor dramatically upregulated the expression level of Bax and cleaved caspase 3, while miR-484 mimics strikingly downregulated these cell apoptosis-related proteins (Figure 2b). Furthermore, flow cytometry assay showed that effects of OGD treatment on murine cortical neurons could be remarkably enlarged by miR-484 inhibition, as evidenced by increased apoptotic cells with miR-484 inhibitor transfection. However, the effects of OGD treatment on murine cortical neurons could also be alleviated by miR-484 overexpression, as evidenced by decreased apoptotic cells with miR-484 mimics transfection (Figure 2c). These results indicate that the survival and apoptosis of OGD-treated neurons could be enlarged or reversed by downregulating or upregulating the level of miR-484.

## MiR-484 can target and regulate BCL2L13 expression level

**Figure 3.**
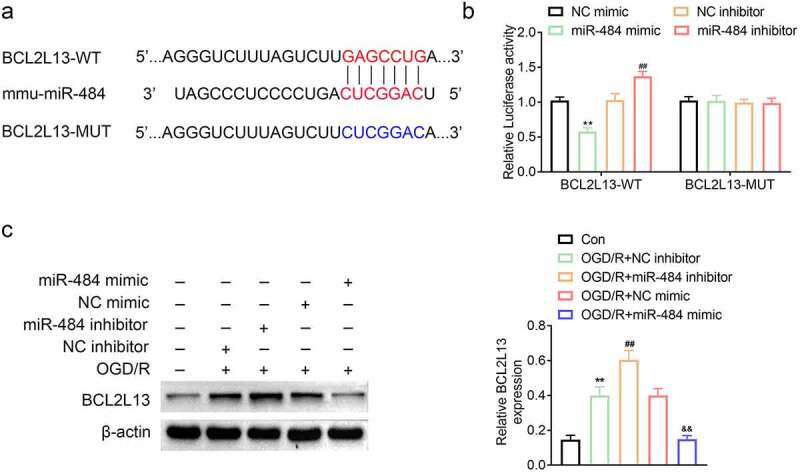
**MiR-484 directly regulated BCL2L13 expression by binding to the 3ʹ-UTR of BCL2L13**. (a) Forecast of miR-484 binding sites on target gene BCL2L13 by TargetScan. (b) Dual-luciferase assays were carried out after cells were co-transfected BCL2L13-WT or BCL2L13-MUT with miR-484 inhibitor, NC inhibitor (control), miR-484 mimics, and NC mimics (control), respectively. (c) The protein expression levels of BCL2L13 in cells were determined by western blot. β-actin was applied as the endogenous reference genes. Data were presented as the mean ± SD with three independent experiments. **p < 0.01 versus control group, ^##^*p* < 0.01 versus OGD treated + NC inhibitor group, and ^&&^*p* < 0.01 versus OGD treateda + NC mimics group

To explore the biomechanism of miR-484 in murine cortical neurons, the mRNA binding sites were further anticipated in TargetScan (http://www.targetscan.org/vert_71/). The results indicated that BCL2L13 mRNA was a binding target of miR-484. The anticipated 3ʹ-UTRs of BCL2L13 mRNA binding to miR-484 were presented (Figure 3a). In order to investigate whether BCL2L13 was a potential target of miR-484, BCL2L13 WT, and MUT fragments were cloned downstream of the fireﬂy luciferase coding region. The results of this study indicated that the overexpression of miR-484 notably brought down the luciferase activity of the BCL2L13-WT reporter gene, but had no effect on the BCL2L13-MUT. Meanwhile, the knockdown of miR-484 dramatically brought up the luciferase activity of the BCL2L13-WT reporter gene but had no effect on the BCL2L13-MUT control (Figure 3b). To further prove whether miR-484 regulated BCL2L13 expression, the expression levels of BCL2L13 protein was detected in the five types of cells described above. The western blot assay results showed that miR-484 inhibitor obviously promoted BCL2L13 protein expression compared with that in the NC inhibitor group. However, miR-484 mimics significantly inhibited BCL2L13 protein expression compared with that in NC mimics group (Figure 3c). These data showed that BCL2L13 is a target of miR-484 and miR-484 represses BCL2L13 protein expression level.

## Overexpression of BCL2L13 reversed the effect of miR-484 in neurons following OGD

**Figure 4.**
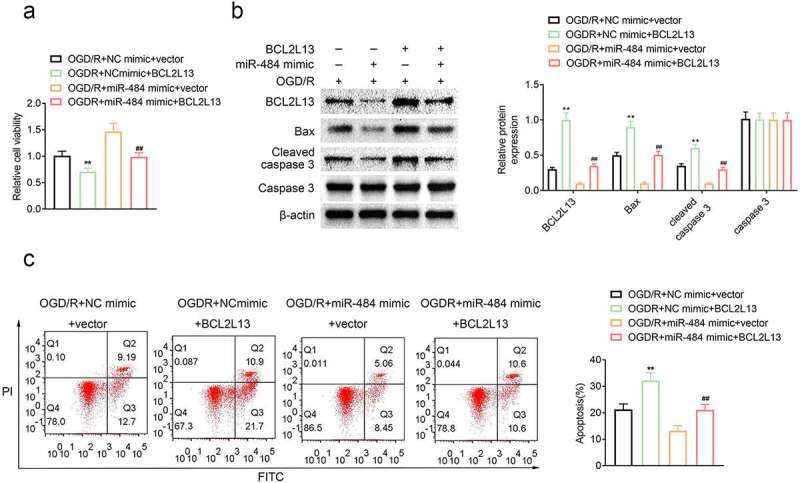
**Overexpression of BCL2L13 reversed the effect of miR-484 in neurons following oxygen-glucose deprivation (OGD)**. (a) CCK8 assay was applied to determine cell viability for each group, including OGD treated + NC mimics + vector group, OGD treated + miR-484 mimics + vector group, OGD treated + NC mimics + BCL2L13 group, and OGD treated + miR-484 mimics + BCL2L13 group. (b)Western blot was applied to detect cell apoptosis-related proteins for each group. (c) Flow cytometry analysis was applied to detect apoptosis for each group. Data were presented as the mean ± SD with three independent experiments. *p < 0.05 and **p < 0.01 versus control group and ^#^*p* < 0.05 and ^##^*p* < 0.01 versus OGD treated + miR-484 mimics + vector group

To explore the role of miR-484-targeted BCL2L13 in the survival and apoptosis of OGD-treated neurons, murine cortical neurons were transfected with NC mimics + vector (control), miR-484 mimics + vector, NC mimics + BCL2L13, and miR-484 mimics + BCL2L13, respectively. Then, cell viability was assessed in each group, and the results revealed that the cell viability of neurons was reduced compared to the NC mimics + vector control group while BCL2L13 overexpressed in neurons. Meanwhile, the cell viability of neurons was enhanced compared to the NC mimics + vector control group while miR-484 overexpressed in neurons as proved previously. However, the cell viability of neurons was reversed while miR-484 and BCL2L13 overexpressed in neurons (Figure 4a). In addition, cell apoptosis-related proteins were also examined by western blot in each group, and the results confirmed that overexpression of BCL2L13 obviously upregulated the expression level of Bax and cleaved caspase 3, while overexpression of miR-484 significantly downregulated BCL2L13 and these cell apoptosis-related proteins. However, the expression level of BCL2L13, Bax, and cleaved caspase 3 was reversed, while miR-484 and BCL2L13 overexpressed in neurons (Figure 4b). Furthermore, flow cytometry assay showed that BCL2L13 promoted apoptosis, as evidenced by increased apoptotic cells with overexpression of BCL2L13. Meanwhile, overexpression of BCL2L13 in miR-484 transfected neurons could also reverse the apoptotic level than the low apoptotic level group which transfected with the miR-484 (Figure 4c). These results proved that the effect of miR-484 in the survival and apoptosis of OGD-treated neurons could be reversed by the overexpression of BCL2L13.

## Overexpression of miR-484 alleviated cerebral ischemia/reperfusion injury in mice

**Figure 5.**
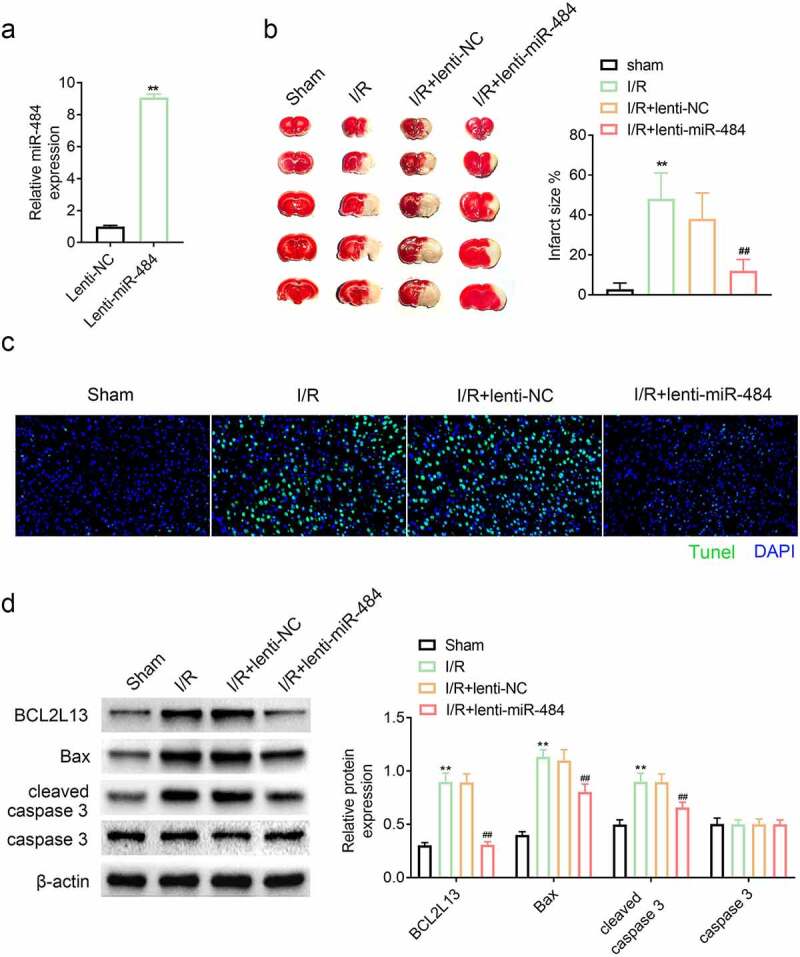
**Overexpression of miR-484 alleviated cerebral ischemia/reperfusion injury in mice**. (a) qPCR was applied to determine miR-484 expression level for each group, including lentiviral miR-484 and lentiviral NC vectors group. (b) Cerebral infarction volume was assessed to check protective effect of miR-484 on cerebral ischemia/reperfusion injury for each group. (c) Tunel assay was applied to determine cell apoptosis for each group. (d) Western blot was applied to detect cell apoptosis-related proteins for each group. **p < 0.01 versus sham-operated group and ^##^*p* < 0.01 versus lentiviral NC group

In order to stably overexpress miR-484 *in vivo*, the constructed lentivirus was injected intravenously and its expression level was confirmed by qPCR. The results confirmed that the miR-484 expression level was dramatically higher than that of the lentiviral NC vectors, indicating that miR-484 is stably overexpressed (Figure 5a). The protective effect of miR-484 on cerebral ischemia/reperfusion injury was evaluated by assessment of cerebral infarction volume and neuron apoptosis in mice after ischemia administration. The cerebral infarction volume results revealed that ischemia administration dramatically enhanced cerebral infarction volume compared with those in the sham-operated group. However, overexpression of miR-484 strikingly reduced cerebral infarction volume compared with those in the lentiviral NC group (Figure 5b). Tunel assay result also hinted that ischemia administration markedly increased Tunel–positive neurons that of the sham-operated group while overexpression of miR-484 dramatically decreased Tunel–positive neurons that of lentiviral NC group (Figure 5c). In addition, cell apoptosis-related proteins were also examined by western blot in each group, and the results confirmed that ischemia administration obviously upregulated the expression level of BCL2L13, Bax, and cleaved caspase 3 while overexpression of miR-484 significantly downregulated BCL2L13 and these cell apoptosis-related proteins, which is consistent with the results of previous experiments (Figure 5d). These results hinted that overexpression of miR-484 alleviated cerebral ischemia/reperfusion injury-induced neuronal apoptosis in mice by targeting apoptosis facilitator BCL2L13.

## Discussion

Recently, some studies have suggested that neuronal apoptosis was considered as one of the main factors of cerebral ischemia/reperfusion injury [19]. The pathological changes in neurons are accurately mediated by extracellular mechanical and intracellular molecular signals. Understanding the molecular regulatory mechanism of neuronal apoptosis under the CIR may provide the novel therapeutic targets for CIR. However, the molecular regulatory mechanism of neurons fate determination under the cerebral ischemia/reperfusion injury remains poorly understood.

MiRNAs have been shown to play vital roles in modulating cell growth and apoptosis processes [20,21]. Many miRNAs are highly expressed in the nervous system, contributing to the maintenance of normal tissue functions and homeostasis [22]. Moreover, many miRNAs are implicated in cell apoptosis processes of neurons, and disorders of miRNAs play a vital role in CIR [23–25]. Specifically, the relationship between long non-coding RNA MEG3 and CIR in neurons was confirmed that they were regulated by miR-485/AIM2 axis [26]. Recently, studies have also found that miR-224-3p participated in the alleviation of apoptosis from CIR in neurons by targeting FIP200 [27]. Moreover, miR-199a was found to attenuate CIR via activating AMPK signaling pathway [28]. However, the specific molecular regulatory mechanism of cell apoptosis under the cerebral ischemia/reperfusion injury of neurons by miRNAs requires further investigation. This study disclosed that the expression level of miR-484 is lower in neurons under the CIR, and the cell viability and apoptosis of neurons could be regulated by upregulating or downregulating the expression level of miR-484. These results hint that miR-484 may act as an inhibiting factor in neuronal apoptosis under the cerebral ischemia/reperfusion injury.

A growing number of researches confirmed that miRNAs carry out their functions by regulating the expression of target mRNAs [29]. A previous study proved that upregulation of miR-496 could decrease cerebral ischemia/reperfusion injury via negatively regulating BCL2L14 signaling pathway [30]. Moreover, miR-34b was reported to participate in protecting against cerebral ischemia/reperfusion injury in rats via targeting Keap1 signaling pathway [31]. In addition, LncRNA MALAT1 was reported to mediate CIR via miR-145/regulate the AQP4 axis [32]. Another novel discovery of this study was that BCL2L13 is a target of miR-484. BCL2L13 belongs to a gene family which encodes a mitochondrially-localized protein. These proteins have been involved in several developmental processes and overexpression of the encoded protein results in apoptosis [8]. Here, the fact that miR-484 could target BCL2L13 was demonstrated by predicting the mRNA binding sites in TargetScan and performing the dual-luciferase assays. Moreover, high expression levels of miR-484 dramatically suppressed BCL2L13 protein expression while low expression levels of miR-484 notably promoted BCL2L13 protein expression. Furthermore, the results proved that miR-484 enhanced cell viability and inhibited cell apoptosis of neurons while this effect could be abrogated by BCL2L13 overexpression. Similarly, overexpression of miR-484 significantly downregulated BCL2L13 and cell apoptosis-related proteins.

## Conclusion

In conclusion, in this study, the fact that miR-484 would be down-regulated in neurons following OGD. Moreover, miR-484 increased viability and inhibited apoptosis of neurons following OGD. Meanwhile, miR-484 regulates the neuronal apoptosis through BCL2L13. Finally, overexpression of BCL2L13 could abolish the effect of miR-484-suppressed cell apoptosis, and overexpression of miR-484 significantly downregulated BCL2L13 and cell apoptosis-related proteins. All these results indicate that miR-484 alleviates cerebral ischemia/reperfusion injury-induced neuronal apoptosis in mice by targeting apoptosis facilitator BCL2L13.

## Data Availability

All data generated or analyzed during this study are included in this published article.

## Highlight

1.The expression level of miR-484 was down-regulated in neurons following oxygen-glucose deprivation (OGD).

2.MiR-484 increased viability and inhibited apoptosis of neurons following OGD.

3.MiR-484 can target and regulate BCL2L13 expression level.

4.Overexpression of BCL2L13 reversed the effect of miR-484 in neurons following OGD.

5.Overexpression of miR-484 alleviated cerebral ischemia/reperfusion injury in mice.
